# Gastric Glomus Tumor: An Uncommon Source for an Acute Upper GI Bleed

**DOI:** 10.1155/2018/7961981

**Published:** 2018-05-13

**Authors:** Douglas Morte, Jason Bingham, Vance Sohn

**Affiliations:** Department of General Surgery, Madigan Army Medical Center, 9040 Fitzsimmons Drive, Tacoma, WA 98431-1100, USA

## Abstract

**Background:**

Glomus tumors are uncommon mesenchymal neoplasms originating from modified smooth muscle cells in the glomus body. They are generally small, solitary lesions found in the distal extremities. Rarely, involvement in the abdominal viscera can occur. In such cases, hematemesis/melena and epigastric discomfort are the most common initial symptoms. Although gastric glomus tumors can demonstrate malignant behavior, criteria for identifying malignant potential have yet to be established.

**Case Presentation:**

We present a rare case of gastric glomus tumor in an otherwise healthy 41-year-old female. The patient initially presented with a significant upper GI bleed requiring a 4 U PRBC transfusion for stabilization. An upper endoscopy with endoscopic ultrasound identified an ulcerated, submucosal mass thought to be consistent with GI stromal tumor (GIST). Once clinically stable, she was scheduled for elective resection. However, prior to resection she experienced a second hemodynamically significant upper GI bleed and underwent emergent laparotomy with distal gastrectomy. Pathologic examination revealed a 3 cm glomus tumor.

**Conclusion:**

Gastric glomus tumors are rare solitary submucosal tumors for which preoperative diagnosis is challenging and can be confused with a GIST. Local resection with negative margins is the preferred treatment and the exact diagnosis relies heavily on histopathological examinations. Currently, there are no clear guidelines regarding the staging and malignant potential of glomus tumors of the stomach.

## 1. Background

Glomus tumors are rare mesenchymal neoplasms that arise from modified smooth muscle cells within the glomus body. They are typically small, solitary tumors and account for approximately 2% of all soft tissue tumors [1]. The glomus body (or glomus apparatus) consists of arteriovenous shunts surrounded by connective tissue and is involved in body temperature regulation. They exist throughout the body but are in highest concentration in the distal extremities. Not surprisingly, the vast majority of glomus body neoplasms are therefore also found in the extremities. Visceral involvement has been described but is exceedingly rare and comprises less than 1% of all gastrointestinal soft tissue tumors [2–6]. The most common site for gastrointestinal involvement is the stomach, specifically the gastric antrum [7]. We present the case of a rare gastric glomus tumor which presented with massive upper gastrointestinal hemorrhage.

## 2. Case Presentation

**Figure 1:**
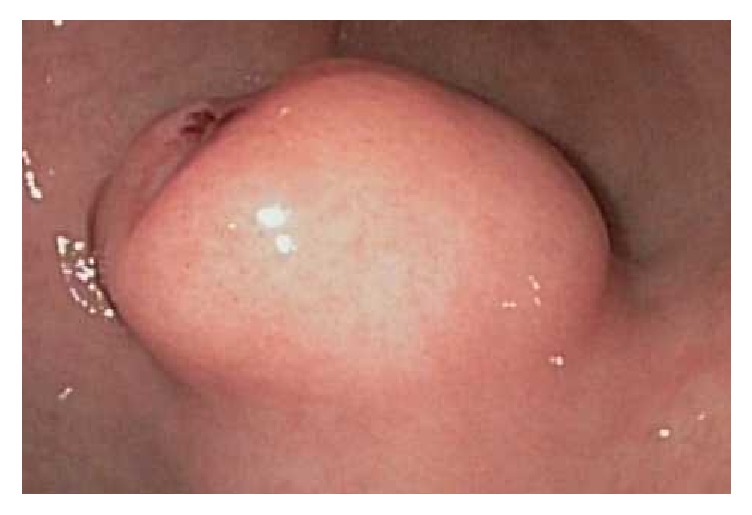
Ulcerated gastric glomus tumor, gross appearance.

**Figure 2:**
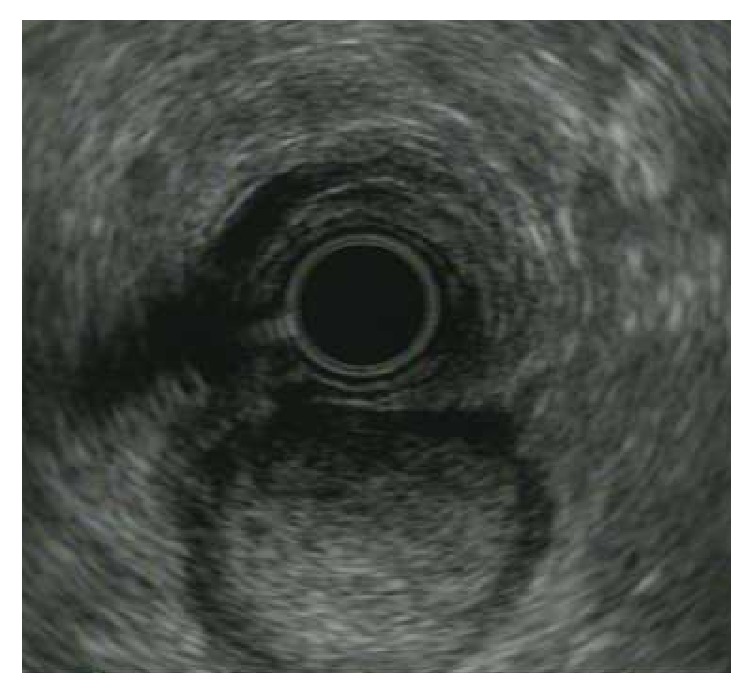
Ultrasound image of glomus tumor.

**Figure 3:**
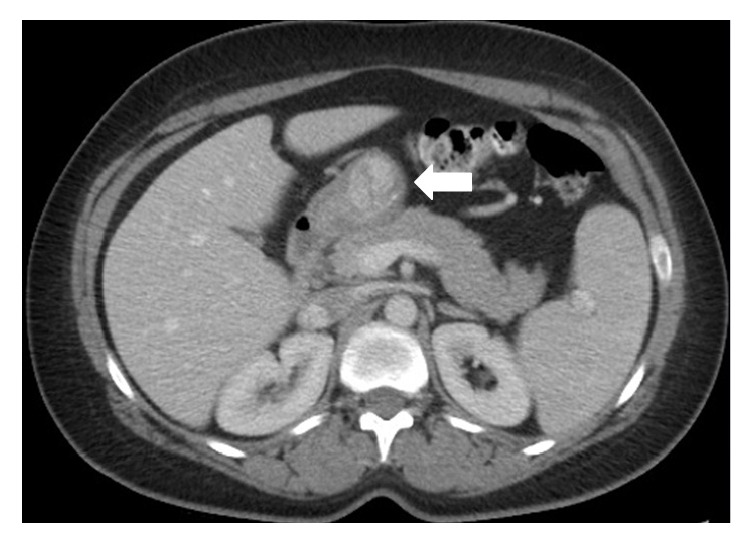
CT image of glomus tumor. The arrow is pointing to a hyperenhancing, prepyloric, submucosal mass.

The patient is an otherwise healthy 41-year-old female who initially presented with a hemodynamically significant, but self-limited, upper GI bleed. She required 4 units of packed red blood cells in total for stabilization. An upper endoscopy was performed which showed an ulcerated gastric mass (Figure 1). A subsequent endoscopic ultrasound revealed a 3 cm well-circumscribed and homogeneous submucosal lesion (Figure 2). CT scan confirmed the 3.0 × 2.9 × 2.7 cm hyperenhancing, prepyloric, submucosal mass with no radiographic evidence of metastasis (Figure 3).

Based on the above imaging findings, the diagnosis was thought to be most consistent with gastrointestinal stromal tumor (GIST) and the patient was scheduled for elective resection. However, on the day prior to her scheduled resection she experienced recurrent upper GI bleeding with hemodynamic instability. An emergent exploratory laparotomy was performed and the antral mass was identified along the greater curvature of the stomach just proximal to the pylorus. It was determined that a simple wedge resection would have likely resulted in gastric outlet obstruction. Thus, a distal gastrectomy with roux-en-y reconstruction was performed. She subsequently had an uneventful hospital course and was discharged home 5 days postoperatively.

**Figure 4:**
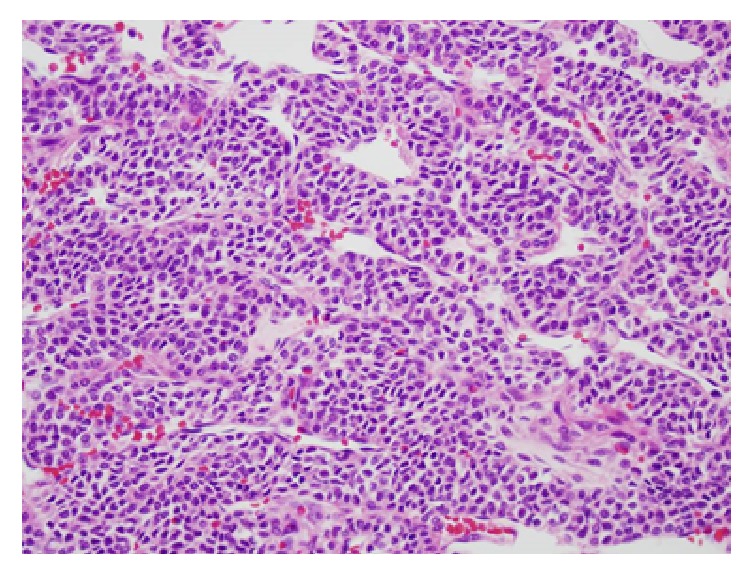
Histologic image of glomus tumor.

**Figure 5:**
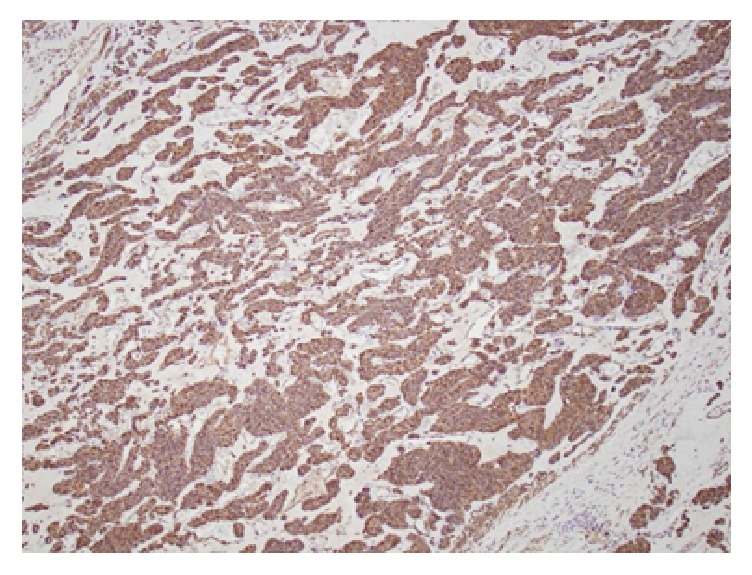
Immunohistochemical staining of glomus tumor, synaptophysin positive.

Interestingly, on pathologic examination the tumor was shown to be a 3 cm, low-grade gastric glomus tumor. All surgical margins were negative. Tumor histology demonstrated a low-mitotic rate (2 mitotic figures per 50 high power field) with no nuclear atypia or lymphovascular invasion (Figure 4). Immunohistochemical staining was notable for positive expression of synaptophysin and smooth muscle actin with absence of CD 117 and CD 34 expression (Figure 5).

## 3. Discussion

The first case of gastric glomus tumor was described in 1951 with sparse reports in the literature since that time [8]. In one of the largest series to date, Miettinen and colleagues described 32 cases of glomus tumors of the GI tract that were referred to the Armed Forces Institute of Pathology over an 18-year period [7]. They found a 3 : 1 female predominance with a median age of presentation of 55 years, although adults of all ages were affected. Despite nuclear atypia and vascular invasion being common on histologic specimen, tumors demonstrated mostly benign clinical behavior. At 50 months follow-up, all but one patient demonstrated a benign course. One patient died of metastatic disease to the liver. This patient's tumor was histologically indistinct from tumors which demonstrated benign behavior, with a low-mitotic rate and the presence of nuclear atypia and vascular invasion. The authors concluded that although gastric glomus tumors have an overall good prognosis, there is a small, unpredictable potential for malignant behavior.

Gastric glomus tumors share many features with other gastric submucosal lesions, making preoperative diagnosis difficult. They are often initially misdiagnosed as the more common GIST, only to be definitively diagnosed by immunohistochemistry on final pathologic specimen. Patients are typically symptomatic at time of presentation, with gastrointestinal bleeding and epigastric discomfort being common [9]. Although endoscopic fine needle aspiration has been described for successful preoperative diagnosis [10], this is usually of low yield given the intramural nature of the tumor. Moreover, the safety and efficacy of this approach have not been established and there is currently no proven method of diagnosis prior to resection.

Postoperative microscopic examination typically demonstrated round, uniform glomus cells within a hypervascular surrounding tissue. Immunohistochemical staining is characteristic for the expression of the mesenchymal markers synaptophysin, smooth muscle actin, laminin, and vimentin. Noticeably absent is CD 117 expression, thus distinguishing glomus tumor from the more common GIST [11]. CD 34 expression is somewhat less reliable but more likely be absent in glomus tumor compared to stromal tumors.

Given the rarity of disease, there are currently no established guidelines for the staging, optimal treatment, or clinical follow-up for these tumors. The diagnostic criteria for malignant gastric glomus tumor remains controversial. Tumor size, necrosis, mitotic activity, and atypical mitotic features are generally accepted as factors suggesting malignant propensity. Interestingly, both nuclear atypia and lymphovascular invasion are common findings and do not necessarily indicate malignant potential. It has been suggested that size > 5 cm is more predictive of malignant behavior than mitotic rate or nuclear atypia [1]. Treatment generally involved wedge resection with negative margins; however distal gastrectomy with reconstruction should be considered with tumors near the pylorus to avoid gastric outlet obstruction. Long-term follow-up is required given the rare malignant potential; however no consensus guidelines currently exist.

## 4. Conclusion

Gastric glomus tumors are rare solitary submucosal tumors with a generally good prognosis that may present as an acute GI bleed. They occur in adults of all ages with a significant female predominance and are typically located in the gastric antrum. Preoperative diagnosis is challenging and often impossible. Given the rarity of these tumors, there are currently no established guidelines for staging, treatment, or follow-up. However, local resection with negative margins is generally considered the treatment of choice. Long-term surveillance is recommended given the small but unpredictable potential for malignant behavior.

## Abbreviations

GI: Gastrointestinal
H&E: Hematoxylin and eosin
GIST: Gastrointestinal stromal tumor.
